# Anti-inflammatory effects of yeast-derived vacuoles on LPS-induced murine macrophage activation

**DOI:** 10.1128/spectrum.01466-23

**Published:** 2023-09-25

**Authors:** Su-Min Lee, Yang-Hoon Kim, Jiho Min

**Affiliations:** 1 Graduate School of Semiconductor and Chemical Engineering, Jeonbuk National University, Jeonbuk, South Korea; 2 School of Biological Sciences, Chungbuk National University, Cheongju, South Korea; University of Mississippi, University, Mississippi, USA

**Keywords:** *S. cerevisiae*-derived vacuole, macrophage, lipopolysaccharide, anti-inflammatory

## Abstract

*Saccharomyces cerevisiae* is a single-celled fungal microorganism. *S. cerevisiae*-derived vacuoles are closely related to mammalian lysosomes, which play a role in the degradation of macromolecules by various hydrolytic enzymes. This study evaluated the anti-inflammatory efficacy of *S. cerevisiae*-vacuoles by inhibiting inflammatory mediators induced by lipopolysaccharide (LPS). The results showed that treatment with 5, 10, and 20 µg/mL of *S. cerevisiae*-derived vacuoles almost completely inhibited the LPS-induced expression of iNOS protein and mRNA. Moreover, vacuoles significantly reduced the mRNA expression of tumor necrosis factor alpha (TNF-α), interleukin 1 beta (IL-1β), and interleukin 6 (IL-6) in LPS-stimulated macrophages compared to the control cells. The immunofluorescence analysis confirmed that *S. cerevisiae*-derived vacuoles inhibited the translocation of nuclear factor kappa-light-chain-enhancer of activated B cells (NF-κB) in LPS-stimulated cells. Taken together, the treatment with *S. cerevisiae*-derived vacuoles alone activated macrophages, but LPS-activated macrophages modulated pro-inflammatory mediators by downregulating the NF-κB pathway. These results suggest that *S. cerevisiae*-derived vacuoles may have therapeutic potential in the treatment of inflammatory diseases. In conclusion, our study provides new insights into the immunomodulatory effects of *S. cerevisiae*-derived vacuoles and their potential as a novel anti-inflammatory agent.

**IMPORTANCE:**

This study investigates the potential of using vacuoles derived from the yeast *Saccharomyces cerevisiae* as a new anti-inflammatory therapy. Inflammation is a natural response of the immune system to invading pathogens, but when it is dysregulated, it can lead to chronic diseases. The researchers found that treating macrophages with vacuoles significantly reduced the production of pro-inflammatory cytokines and iNOS, markers of inflammation when they were stimulated with lipopolysaccharide. The study also showed that vacuoles inhibited the NF-κB signaling pathway, which is involved in the induction of pro-inflammatory cytokines in macrophages. These findings suggest that *S. cerevisiae*-derived vacuoles may have potential as a new therapeutic agent for regulating the inflammatory response in various diseases. Further studies are needed to evaluate the efficacy and safety of vacuoles *in vivo* and to elucidate the underlying mechanisms of their anti-inflammatory effects.

## INTRODUCTION

*Saccharomyces cerevisiae* (*S. cerevisiae*) is a single-celled fungal microorganism that was first discovered on malt in 1837. It has been used extensively in winemaking, baking, and brewing. The cells are round to ovoid and 5–10 µM in diameter. *S. cerevisiae* is one of the most intensively studied eukaryotic model organisms in molecular and cellular biology and closely resembles the model bacterium *Escherichia coli* (1). Vacuoles are membrane-bound organelles found in *S. cerevisiae* that are closely related to mammalian lysosomes. They play a role in the degradation of macromolecules by various hydrolytic enzymes (2). The *S. cerevisiae*-derived vacuole contains over 60 hydrolytic enzymes, making this organelle essential for degrading cellular lipids and cytoplasmic proteins (3, 4). Vacuoles also play a vital role in pH and ion homeostasis, innate immune pathways, stress signaling responses, and function as an ion storage compartment (5). The misregulation of vacuole function has many implications in human diseases, especially neurodegenerative diseases.

Lipopolysaccharide (LPS) is the most prevalent antigen on the surface of Gram-negative bacteria, and it usually triggers macrophage differentiation into the M1 type (6). Structurally, LPS is made up of lipid A, a core oligosaccharide, and an O-side chain. Lipid A, among the LPS components, is highly immunogenic and catalyzes the transfer of LPS to CD14, which is recognized by lipopolysaccharide-binding protein (7). The myeloid differentiation protein 2 (MD-2)-toll-like receptor 4 (TLR4) complex initiates endotoxin signaling and interacts with CD14, which lacks a cytoplasmic domain and transmembrane. TLR4 present on the surface of macrophages, monocytes, dendritic, and B cells recognizes LPS. The TLR4-MD-2-LPS complex is formed, and both MyD88-dependent and MyD88-independent signaling pathways can recruit downstream adapter proteins to upregulate inflammatory gene expression. The MyD88-dependent pathway recruits IRAK4 (IL-1 receptor-associated kinase-4), IRAK1, and IRAK2, which are phosphorylated, separated, and bound to TRAF6 (8). TRAF6 activates the TAK1 complex, which induces the IKKα/IKBKB complex, which then phosphorylates IκB, which is ubiquitinated and degraded. The MyD88-independent pathway recruits TIR domain-bearing TRAM and TRIF and activates the transcription factor IRF3, which regulates transcription entering the nucleus (9, 10). Therefore, LPS is recognized as a potent stimulator of the immune system and has been widely used as a model for the study of inflammation and immunity (11, 12).

In our previous studies, we investigated the immunomodulatory effects of vacuoles isolated from *S. cerevisiae* and demonstrated their potential as a novel immunomodulatory agent. The studies showed that vacuole treatment increased phagocytosis and activated various immune responses, including toll-like receptor 2/4-mediated phagocytosis and immune response activation in macrophages. Vacuole treatment also activated NF-κB and MAPK signaling pathways and produced pro-inflammatory cytokines (13, 14). These findings suggest that *S. cerevisiae*-derived vacuoles have potential as a novel immunomodulatory agent. Based on these studies, it is possible that treating LPS-stimulated macrophages with vacuoles could potentially modulate the pro-inflammatory response. Specifically, the vacuoles may inhibit the LPS-induced expression of inflammatory mediators such as TNF-α, IL-1β, and IL-6, as well as iNOS protein and mRNA. Additionally, the vacuoles may inhibit the NF-κB pathway, which is a key regulator of inflammation. However, further research is necessary to determine the specific effects of vacuole treatment on LPS-stimulated macrophages.

Therefore, the aim of this study was to investigate the effects of treating mouse macrophages stimulated with LPS *in vitro* with vacuoles isolated from *S. cerevisiae*. Our findings provide important insights into the inhibitory effects of LPS-induced pro-inflammatory cytokines and inflammatory mediators in yeast-derived vacuoles and their potential mechanisms of action, thereby underscoring the significance of our research in the field of immunomodulation. Furthermore, our study focuses on the potential of *S. cerevisiae*-derived vacuoles as a novel immunomodulatory agent for modulating the LPS-induced pro-inflammatory response. By elucidating the mechanisms by which these vacuoles modulate the immune response, our research may contribute to the development of new therapeutic strategies for inflammatory disorders, providing a potential avenue for treating a wide range of diseases.

## MATERIALS AND METHODS

### Reagents and antibodies

*E. coli* O111:B4 lipopolysaccharides (LPS) and dexamethasone were purchased from Sigma Aldrich (St. Louis, MO, USA). The primary antibodies used in this study, including anti-mouse iNOS (1:3,000 dilution), NF-kB p65 antibodies (1:200 dilution), and Alexa Fluor 488 anti-NF-kB p65 antibody, were obtained from Abcam (Cambridge, UK). The anti-β-actin antibody (1:1,000 dilution) was sourced from Cusabio (Wuhan, China). Horseradish peroxidase (HRP)-labeled goat anti-mouse IgG and HRP-labeled goat anti-rabbit IgG were purchased from Cell Signaling Technology (Beverly, MA, USA). Enzyme immunoassay reagents for the detection of TNF-α and IL-6 cytokines were obtained from BD Biosciences (San Jose, CA, USA).

### Cell culture

The murine macrophage cell line RAW 264.7 cells were obtained from the Korean Cell Line Bank (470071) and cultured in Dulbecco’s modified Eagle’s medium (DMEM) supplemented with 10% fetal bovine serum (FBS) and penicillin-streptomycin (100 U/mL and 100 µg/mL) at 37°C in a humidified 5% CO_2_ incubator.

### Isolation of *S. cerevisiae*-derived vacuoles

The vacuoles used in this experiment were extracted in the same manner as described in references (13, 14). The yeast *Saccharomyces cerevisiae* (ATCC 208208) was cultured in yeast extract peptone dextrose (YPD) medium until reaching an optical density (OD)_600_ of 0.8–0.9. After centrifugation, the yeast cells were treated with Tris-SO4 buffer (0.1 M, pH 9.4) containing 10 mM dithiothreitol (DTT) and incubated at 30°C and 90 rpm for 15 minutes to affect the cell walls. Following a second centrifugation, the yeast cell pellet was resuspended in a breaking buffer (20 mM Tris-HCl, pH 7.4; 0.6 M sorbitol; 1 mM phenylmethylsulfonyl fluoride [PMSF]), and cells were subjected to vortexing for a total of 20 minutes (1 minute on/off cycle, repeated 10 times) with glass beads. The cell debris and glass beads were separated from the supernatant by centrifugation at 500 × *g* for 10 minutes. Finally, the microtube containing the supernatant was subjected to further centrifugation at 20,000 × *g* for 30 minutes using a microcentrifuge. The resulting supernatant, after this second centrifugation, can be used for subsequent analyses or experiments.

### Bio-transmission electron microscope

The *S. cerevisiae*-derived vacuoles were analyzed using bio-transmission electron microscope (Bio-TEM; H-7650, Hitachi, Tokyo, Japan) imaging. The obtained vacuoles were diluted 1:100,000 in distilled water (DW). Place a drop of 1 µL diluted vacuole on the grid for 1 minute. After drying the filter paper, stain with 1% uranyl acetate for 3 seconds. The prepared samples were observed using Bio-TEM located at the Center of University-wide Research Facilities at Jeonbuk National University.

### Field emission scanning electron microscopy

The *S. cerevisiae*-derived vacuoles were analyzed using field emission scanning electron microscopy (FE-SEM; SUPRA40VP, Carl Zeiss, Germany) imaging. The obtained vacuoles were diluted 1:100 in DW and subjected to lyophilization for 4 hours, resulting in the formation of a powder. The total volume used for the lyophilization process was 100 µL. The prepared samples were observed using FE-SEM located at the Center of University-wide Research Facilities at Jeonbuk National University.

### Nanoparticle tracking analysis

The size distribution of vacuoles was analyzed using nanoparticle tracking analysis (NTA). The vacuoles obtained were diluted 1:500,000 in DW and analyzed using the Zetaview system (Particle Metrix GmbH, Germany).

### Western blot

RAW 264.7 cells were seeded at a density of 1 × 10^6^ cells/well in a 100 mM dish and incubated for 24 hours. The cells were then starved with DMEM containing 1% FBS, 100 µg/mL streptomycin, and 100 U/mL penicillin for 12 hours. LPS (1 µg/mL) was added to the cells and incubated for 2 hours, followed by treatment with vacuoles at concentrations of 5, 10, and 20 µg/mL for an additional 22 hours. After washing the vacuoles with Dulbecco's phosphate buffered saline (DPBS) three times, the cells were lysed using radioimmunoprecipitation assay buffer (RIPA buffer). The protein concentration of each sample was determined using the Bradford assay and adjusted to 20 µg. The protein samples were separated by molecular weight using 10% SDS-PAGE gel and transferred onto a nitrocellulose membrane. The membrane was blocked with 5% skim milk for 1 hour, and then incubated with primary antibody overnight at 4°C. After washing the membrane with T-TBS (pH 7.4), the secondary antibody was added and incubated at room temperature for 1 hour. The results were expressed as a percentage relative to β-actin.

### Cytokine secretion assays

The murine RAW 264.7 cells were cultured until they reached approximately 90% confluency in a 100 mM dish. Then, 1 × 10^5^ cells were seeded in each well of a 24-well plate and incubated for 24 hours to allow stabilization. Afterward, the cells were starved with DMEM without phenol red, containing 1% FBS, 100 µg/mL streptomycin, and 100 U/mL penicillin, for 12 hours. Following a 2-hour pre-treatment with LPS (1 µg/mL), vacuoles were applied at different concentrations (5, 10, and 20 µg/mL) for 22 hours, and the resulting supernatant was collected. Enzyme-linked immunosorbent assay (ELISA) was performed by adding 50 µL of ELISA diluent to each well of a 96-well plate coated with antibodies, followed by the addition of 50 µL of sample and diluted standards. After a 2-hour incubation at room temperature, the plate was washed five times with washing buffer. Enzyme working reagent was then added and incubated for 30 minutes. Following seven additional washes, 3,3´,5,5´-tetramethylbenzidine (TMB) One-Step Substrate Reagent was added and the plate was incubated for another 30 minutes. Finally, the absorbance at 450 nm was measured after adding 50 µL of stop solution

### Reverse transcription quantitative PCR (RT-qPCR)

Reverse transcription quantitative PCR (RT-qPCR) was used to analyze the effect of vacuolar cytokine levels on LPS-stimulated macrophages. RAW 264.7 cells were seeded at 1 × 10^6^ (cells/well) in a 100 mM dish and starved with serum-free DMEM for 12 hours. The cells were pre-treated with LPS (1 µg/mL) for 2 hours, followed by a 22-hour treatment with vacuoles at concentrations of 5, 10, and 20 µg/mL. Total RNA was extracted from RAW 264.7 cells using the GeneAll Ribospin II kit (GeneAll Biotechnology) following the manufacturer’s instructions. Cytokine primer sequences were analyzed using Table 1; Table S1, and a one-step SYBR Green reagent (AccuPower GreenStar RT-qPCR, Bioneer Co., South Korea) was used. After pre-denaturation at 95°C for 15 minutes, PCR was performed for 45 cycles at a denaturation temperature of 95°C for 15 seconds and an annealing/extension temperature of 60°C for 30 seconds. CFX Maestro software (Bio-Rad Laboratories, Inc., USA) was used for analysis.

**TABLE 1:** Primer sequences for real time-PCR

Genes	Forward primer sequence	Reverse primer sequence
TNF-α	5′-GCCTCTTCTCATTCCTGCTT-3′	5′-CTCCTCCACTTGGTGGTTTG-3′
IL-6	5′-GTTCTCTGGGAAATCGTGGA-3′	5′-GGTACTCCAGAAGACCAGAGGA-3′
iNOS	5′-CACCTTGGAGTTCACCCAGT-3′	5′-ACCACTCGTACTTGGGATGC-3′
COX-2	5′-AGAAGGAAATGGCTGCAGAA-3′	5′-GCTCGGCTTCCAGTATTGAG-3′
GAPDH	5′-CTTTGTCAAGCTCATTTCCTGG-3′	5′-TCTTGCTCAGTGTCCTTGC-3′

### Immunofluorescence staining

To analyze the expression of NF-kB p65, RAW 264.7 cells were cultured on glass coverslips in 6-well plates at a density of 2 × 10^5^ cells/well for 24 hours. After treatment with LPS and vacuoles at different concentrations, the cells were cultured for an additional 24 hours. The cells were fixed using 10% formalin for 15 minutes at room temperature, followed by permeabilization with 0.2% (vol/vol) Triton X-100 in PBS for 30 minutes at room temperature. Blocking was performed with 5% bovine serum albumin (BSA) in PBS for 1 hour at room temperature. The primary antibody against NF-kB p65 was added to the cells and incubated overnight at 4°C in 5% BSA in PBS (dilution 1:200). The cells were then treated with Alexa Fluor 488 anti-NF-kB p65 secondary antibody in 1% BSA (dilution 1:50) in PBS for 2 hours at 37°C. DAPI (5 µg/mL) was added to the cells and incubated at room temperature for 5 minutes for staining. Finally, the cells were observed and imaged using a laser-scanning confocal microscope.

### Data analysis

Each data point was obtained from three independent samples conducted simultaneously for error analysis. The results are reported as means ± standard deviations, and the correlations were determined for several experimental conditions. Data analysis was performed using SigmaPlot (Systat Software, Inc., USA). Statistical significance was determined at a *P* value < 0.05.

## RESULTS

### *S. cerevisiae-*derived vacuole characterization

**Fig 1:**
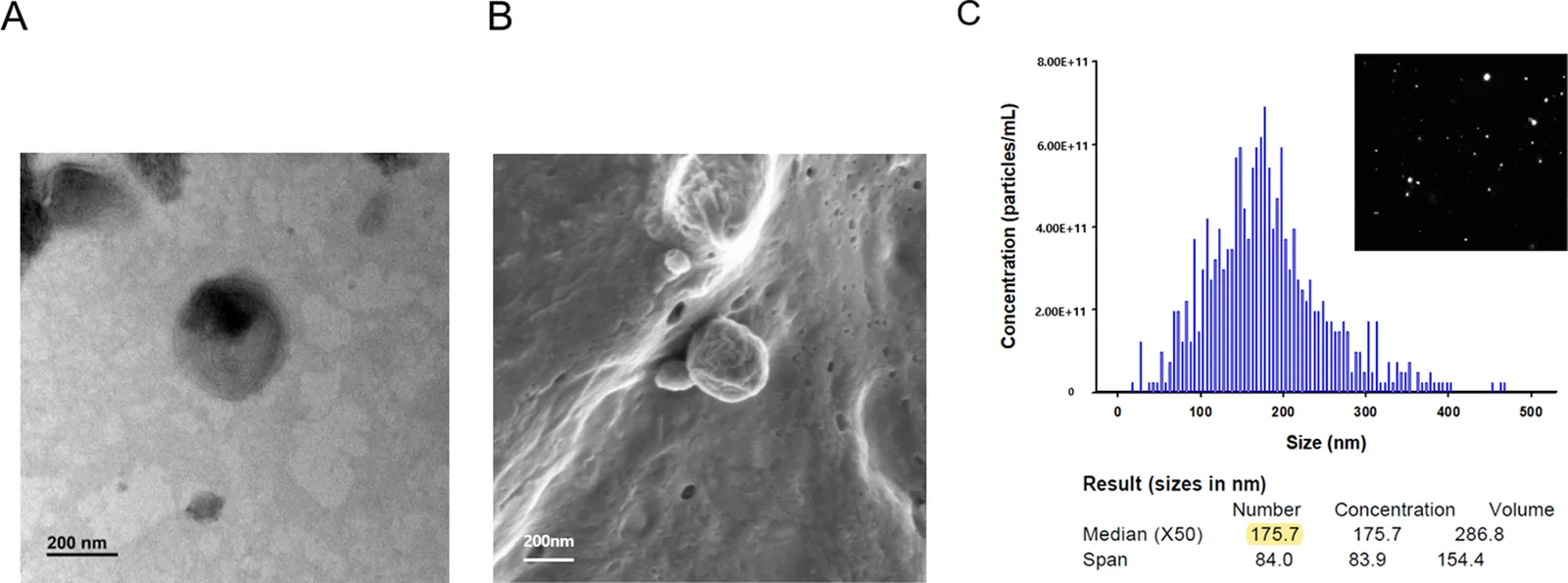
Characterization of *S. cerevisiae*-derived vacuoles. (A) Bio-TEM images of the *S. cerevisiae*-derived vacuoles. (B) FE-SEM images of the vacuoles. (C) Size distribution curve of the vacuoles analyzed by NTA.

The morphology and size distribution of *S. cerevisiae*-derived vacuoles were confirmed using various techniques. Bio-TEM and FE-SEM images (Fig. 1A and B, respectively) showed that the vacuoles had a spherical double membrane structure. Additionally, nanoparticle tracking analysis (Fig. 1C) revealed that yeast-derived vacuoles accounted for 56.0% of the 166.9 nm particle size and 29.3% of the 243 nm particle size, with a peak in the 100–200 nm range and a median diameter of 175.7 nm. These results suggest that the vacuoles used in this study were of appropriate size and morphology for further experimentation.

### Effect of *S. cerevisiae*-vacuole on expressions of iNOS in LPS-stimulated RAW 264.7 cells

**Fig 2:**
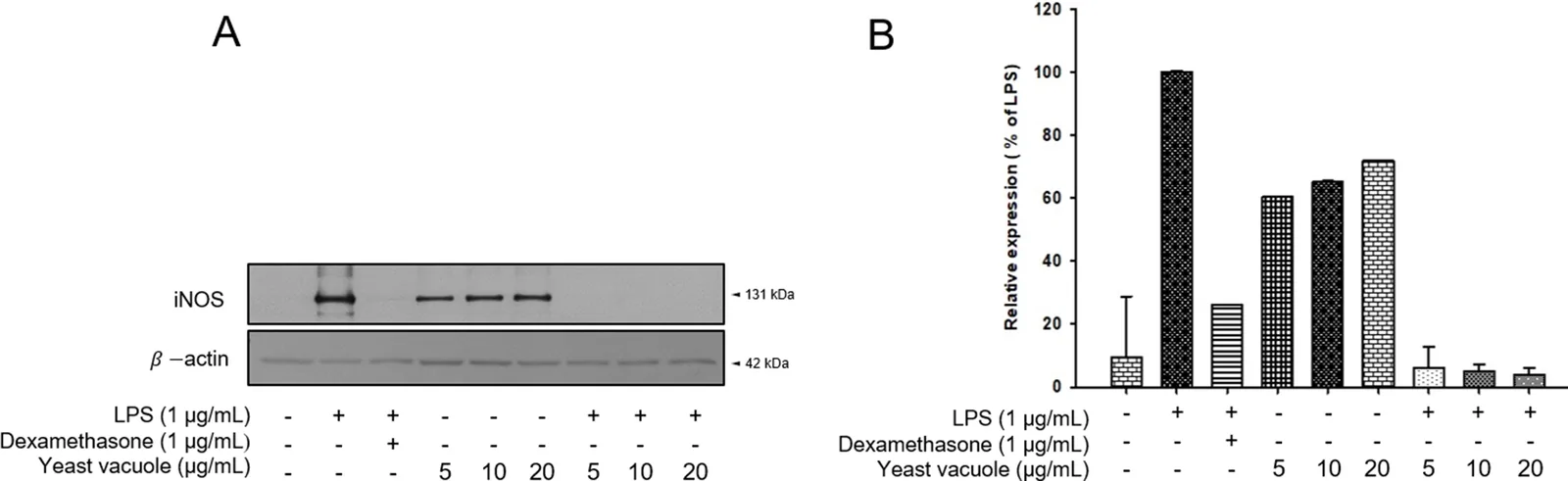
Effect of *S. cerevisiae*-derived vacuoles on the expression of iNOS in LPS-stimulated RAW 264.7 cells. (A) The cells were stimulated with 1 µg/mL LPS for 2 hours at the indicated doses, treated with dexamethasone or vacuoles for 22 hours, and then assessed for iNOS protein using Western blot analysis. (B) The relative ratio of iNOS to β-actin was determined using densitometry, with LPS being employed as a control.

In Fig. 2, we investigated the effect of vacuoles on iNOS protein expression in LPS-stimulated RAW 264.7 macrophages to study the anti-inflammatory mechanism. Cells were pre-treated with 1 µg/mL LPS for 2 hours, followed by treatment with dexamethasone or vacuoles at concentrations of 5, 10, or 20 µg/mL for 22 hours, and the levels of iNOS protein expression were compared. As shown in Fig. 2A, we observed a dose-dependent increase in iNOS protein expression in both LPS-treated and *S. cerevisiae*-derived vacuole-only treated groups. However, treatment of LPS-stimulated RAW 264.7 cells with vacuoles almost completely suppressed the LPS-induced expression of iNOS protein at all concentrations used. These findings suggest that *S. cerevisiae*-derived vacuoles have the potential to modulate the LPS-induced inflammatory response by inhibiting the expression of iNOS protein in macrophages.

### Vacuole extract from yeast inhibits LPS-induced pro-inflammatory cytokines

**Fig 3:**
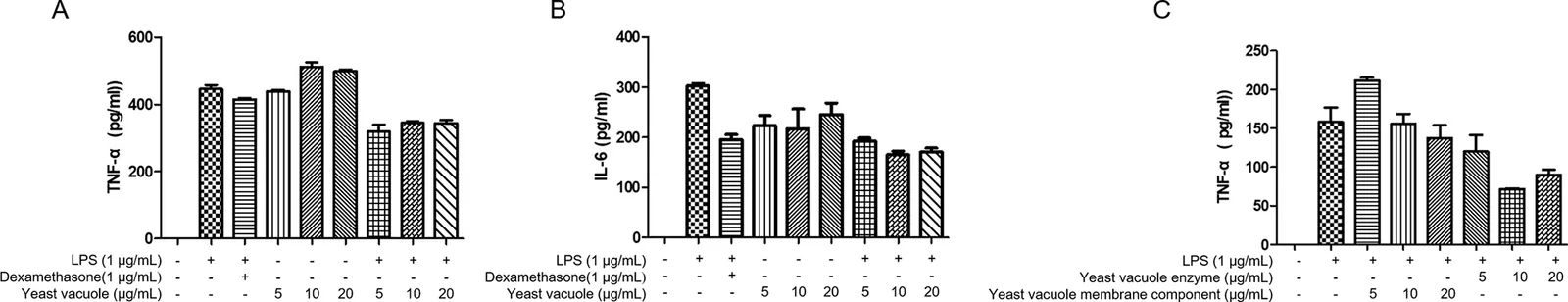
*S. cerevisiae*-derived vacuoles inhibit LPS-induced pro-inflammatory cytokines such as (A) TNF-α and (B) IL-6 protein production. (C) *S. cerevisiae*-derived vacuole enzyme and pellet components change TNF-α cytokine production. Cells were stimulated with 1 µg/mL LPS at the indicated doses for 2 hours, treated with dexamethasone or vacuoles for 22 hours, and then analyzed using ELISA.

The study aimed to investigate the impact of vacuoles on the production of pro-inflammatory cytokines TNF-α and IL-6 in macrophages induced by LPS. As shown in Fig. 3, the levels of TNF-α and IL-6 produced in RAW 264.7 macrophages significantly increased in the LPS-treated group (LPS+) compared to the LPS-untreated group (LPS−). Notably, treatment with *S. cerevisiae*-derived vacuoles at 5 µg/mL after LPS pre-treatment reduced the amount of TNF-α produced by approximately 30% (Fig. 3A), while treatment with vacuoles at 20 µg/mL after LPS pre-treatment reduced the amount of IL-6 produced by about 44% (Fig. 3B). Moreover, upon exposure of TNF- α induced by LPS to membrane proteins and enzymes, components of the vacuole, we observed a significant decrease in the enzyme-treated group (Fig. 3C). These findings suggest that vacuole enzymes derived from *S. cerevisiae* have the potential to modulate the LPS-induced production of pro-inflammatory cytokines in macrophages. However, it is important to note that further research is required to validate and confirm this interpretation.

### Vacuoles modulate inflammatory gene expression in LPS-stimulated macrophages

**Fig 4:**
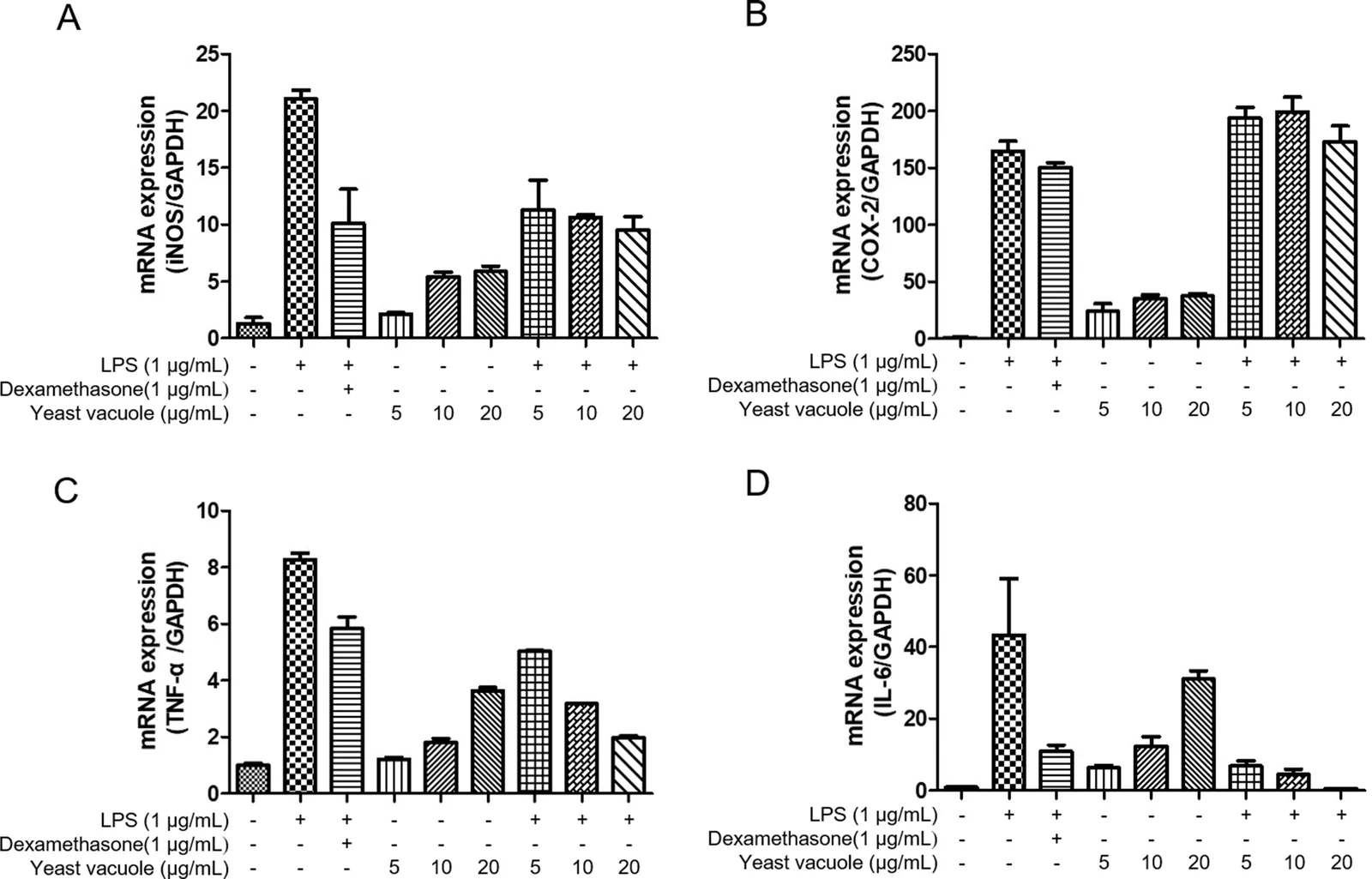
Inhibitory effect of *S. cerevisiae*-derived vacuoles on mRNA expression levels of (A) iNOS, (B) COX-2, (C) TNF-α, and (D) IL-6 in RAW 264.7 cells stimulated by LPS. The cells were stimulated with 1 µg/mL LPS for 2 hours, followed by treatment with dexamethasone or vacuoles for 22 hours. The mRNA levels were measured using reverse transcription quantitative PCR (RT-qPCR). The gene expression levels were normalized to the expression of the housekeeping gene GAPDH.

The effect of *S. cerevisiae*-derived vacuoles on the mRNA levels of iNOS and COX-2 in macrophages induced by LPS was investigated, and the results are presented in Fig. 4. The findings demonstrate that vacuoles effectively downregulate iNOS gene induction, reducing it by approximately 50% compared to the LPS-treated group. However, COX-2 gene expression did not decrease under the same conditions (Fig. 4B). Additionally, the impact of vacuoles on the mRNA levels of TNF-α and IL-6 in RAW 264.7 cells induced by LPS was assessed. As shown in Fig. 4C and D, RT-qPCR analysis revealed that vacuoles reduced LPS-stimulated cytokine mRNA expression. In LPS-stimulated macrophages, vacuoles significantly decreased cytokine mRNA expression in a concentration-dependent manner. TNF-α mRNA expression was reduced by around 70%, and IL-6 was reduced by over 90% at a vacuolar concentration of 20 µg/mL. Although the effect at the protein level was not confirmed, IL-1β mRNA expression (Fig. S1) was reduced by about 80% at a vacuolar concentration of 20 µg/mL. The outcomes suggest that *S. cerevisiae*-derived vacuoles have the potential to regulate the inflammatory response in macrophages at the gene level by effectively downregulating iNOS gene induction and decreasing LPS-stimulated cytokine mRNA expression in a concentration-dependent manner. Furthermore, the impact of *S. cerevisiae*-derived vacuoles on mRNA levels of anti-inflammatory cytokines in LPS-induced macrophages was investigated. The findings revealed a significant upregulation in the expression of anti-inflammatory cytokines such as IL-4 and IL-10 in the group treated with vacuoles compared to the group treated with LPS alone (Fig. S3). To validate the potential of yeast-derived vacuoles as therapeutic agents for modulating inflammatory responses in various diseases, we conducted additional investigations to determine their effects on the regulation of inflammatory cytokines in human cell lines. It was observed that pre-treatment of human lung fibroblasts with vacuoles led to the suppression of pro-inflammatory cytokines induced by hydrogen peroxide (Fig. S4)

### Effect of vacuoles on the translocation of NF-κB p65 in LPS-stimulated RAW 264.7 cells

**Fig 5:**
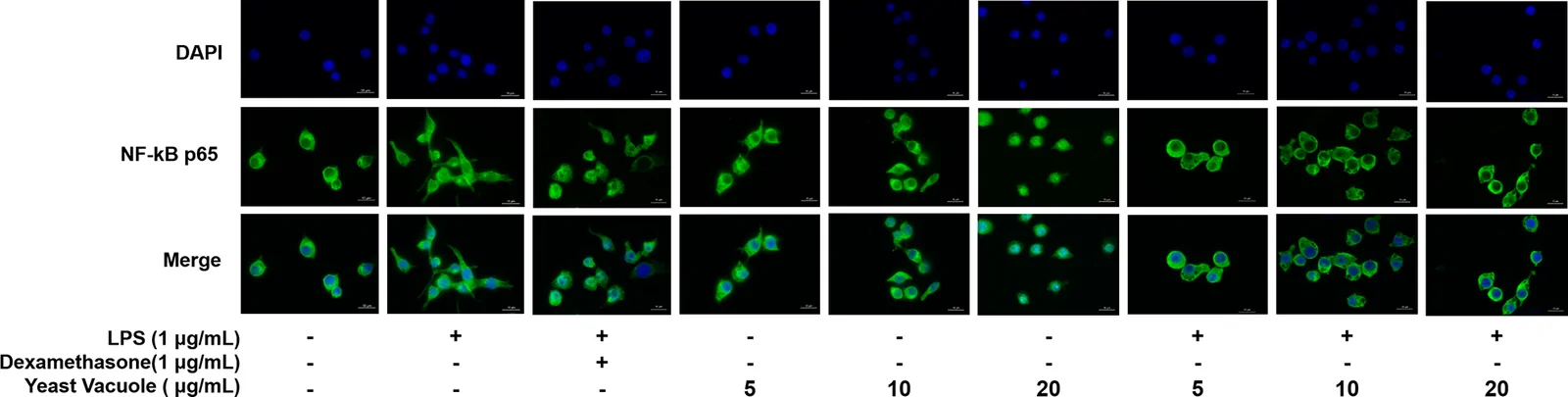
Effect of *S. cerevise*-derived vacuoles on the translocation of NF-κB p65 in LPS-stimulated RAW 264.7 cells. Cells were stimulated with 1 µg/mL LPS, and the nuclear and cytoplasmic localization of p65 was evaluated using an NF-κB p65 monoclonal antibody, a goat anti-rabbit IgG H&L antibody (green), and DAPI nuclear stain (blue). Scale bar = 10 µM.

The study aimed to investigate the effect of vacuoles on the translocation of the p65 subunit of NF-κB from the cytoplasm to the nucleus in RAW264.7 cells. Immunofluorescence analysis revealed that, in unstimulated cells, NF-κB p65 was predominantly located in the cytoplasm, but with LPS stimulation, it translocated to the nucleus. Treatment with vacuoles alone in RAW264.7 cells resulted in nuclear migration and accumulation of NF-κBp65 (Fig. 5). However when vacuoles were added to LPS-stimulated cells, they inhibited NF-κB translocation, as evidenced by weak nuclear p65 staining (Fig. 5). These NF-κB changes were also confirmed in nuclear, cytosol, and whole-cell fractions, and similar patterns were observed (Fig. S2). These results suggest that vacuoles derived from *S. cerevisiae* may inhibit LPS-induced inflammatory responses in macrophages by interfering with NF-κB translocation from the cytoplasm to the nucleus. Our findings provide novel insights into the potential therapeutic applications of vacuoles in modulating immune responses.

## DISCUSSION

*S. cerevisiae*-derived vacuoles are functional analogs of mammalian lysosomes, containing more than 60 different hydrolases that can break down a variety of molecules. Several studies have demonstrated the antibacterial and antiviral effects of vacuoles through the action of these enzymes, which can be enhanced by optimizing the culture conditions (15, 16). Moreover, surface modification of the vacuole membrane to mimic mammalian cell membranes has shown promise for drug delivery applications (17 – 19). Previous studies have shown that treatment with yeast-derived vacuoles alone can induce the upregulation of various immune response mediators, including NO, iNOS, TNF-α, and IL-6, via TLR2/4-mediated pathways in RAW264.7 cells (13, 14). However, the effect of vacuoles on immune response mediators secreted from macrophages activated by LPS has not been reported.

Inflammation is a complex process that plays a protective role against invading pathogens but can also lead to chronic diseases when dysregulated (20 – 22). LPS-induced activation of macrophages triggers the upregulation of nitric oxide and iNOS, which can be used as markers of inflammation, and inhibition of iNOS overproduction is a potential target for anti-inflammatory therapy (23 – 25). In this study, we found that treatment with yeast-derived vacuoles at various concentrations significantly inhibited the LPS-induced expression of iNOS protein and mRNA in macrophages (Fig. 2 and 4A). Similarly, vacuoles reduced the protein and mRNA expressions of TNF-α, IL-1β, and IL-6 in LPS-stimulated macrophages at all concentrations tested (Fig. 3; Fig. 4C and D; Fig. S1). Exposure of LPS-induced pro-inflammatory cytokines to vacuole enzymes and membrane proteins resulted in a significant decrease in the enzyme-treated group, indicating the degradation of these TNF-α by vacuole enzymes (Fig. 3C). These results suggest that vacuolar enzymes derived from *S. cerevisiae* have the potential to affect the production of pro-inflammatory cytokines in macrophages during LPS stimulation. Furthermore, *S. cerevisiae*-derived vacuoles significantly upregulated anti-inflammatory cytokine expression in LPS-induced macrophages compared to LPS treatment alone (Fig. S3). Through additional investigations on human cell lines, yeast-derived vacuoles were found to effectively suppress pro-inflammatory cytokines induced by hydrogen peroxide in human lung fibroblasts (Fig. S4). These cytokines are critical mediators of the inflammatory response, regulating immune cell functions and contributing to various human diseases when dysregulated (22, 26). NF-κB activation is a key step in the induction of pro-inflammatory cytokines in LPS-stimulated macrophages, and vacuoles have been shown to modulate this pathway by interfering with the translocation of NF-κB p65 from the cytoplasm to the nucleus (27). Specifically, treatment with vacuoles alone induced nuclear migration and accumulation of NF-κB p65, while the addition of vacuoles to LPS-stimulated cells inhibited NF-κB translocation, as evidenced by reduced nuclear p65 staining (Fig. 5).

These findings suggest that yeast-derived vacuoles may have potential as a therapeutic agent for regulating the inflammatory response in various diseases. Overall, the study provides important insights into the potential of yeast-derived vacuoles as a therapeutic agent for regulating the inflammatory response in various diseases. However, further studies are needed to fully understand the underlying mechanisms and to evaluate the efficacy and safety of vacuoles *in vivo*.

### Conclusion

In summary, our study investigated the inhibitory effects of LPS-induced inflammatory mediators in mouse macrophage RAW 264.7 cells and found that treatment with *S. cerevisiae*-derived vacuoles significantly reduced the production of pro-inflammatory cytokines and iNOS. Our analysis of the signaling pathway revealed that vacuoles inhibited NF-κB p65 expression and nuclear migration in LPS-induced cells. These findings suggest that *S. cerevisiae-*derived vacuoles can effectively exert anti-inflammatory activity by inhibiting the NF-κB signaling pathway in mouse macrophages. Overall, our study highlights the potential of *S. cerevisiae*-derived vacuoles as a therapeutic agent for regulating the inflammatory response in various diseases. Further studies are needed to investigate the underlying mechanisms and to evaluate the safety and efficacy of vacuole therapy *in vivo*.

## Data Availability

The data that support the findings of this study are available from the corresponding author upon reasonable request. All data, associated methods, and sources of materials are available in the main text.

## SUPPLEMENTAL MATERIAL

Supplemental file 1 spectrum.01466-23-s0001.docx Fig. S1 to S4 and Table S1.

ASM does not own the copyrights to Supplemental Material that may be linked to, or accessed through, an article. The authors have granted ASM a non-exclusive, world-wide license to publish the Supplemental Material files. Please contact the corresponding author directly for reuse.
