# Supplementary material for: Anti-inflammatory effects of yeast-derived vacuoles on LPS-induced murine macrophage activation
Source: Microbiol Spectr. 2023 Sep 25;11(5):e01466-23. doi: 10.1128/spectrum.01466-23 (PMC10580869; doi:10.1128/spectrum.01466-23)
Supplement: Supplemental file 1 — Fig. S1 to S4 and Table S1. [file spectrum.01466-23-s0001.docx]

**FIGURE LEGENDS**

**Figure S1. Inhibitory effect of vacuole on the mRNA level of Pro-inflammatory such as IL-1β in RAW 264.7 cells stimulated by LPS**. Cells were stimulated with 1 μg/ml LPS at the indicated doses for 2 h, then treated with dexamethasone or vacuole for 22 h. The mRNA levels were measured by reverse transcription quantitative polymerase chain reaction (RT-qPCR), DEX: Dexamethasone

**Figure S2. Effect of vacuole on the translocation of NF-κB p65 in the LPS stimulated RAW 264.7 cells for western blots**. Cells were stimulated with 1 μg/ml LPS at the indicated doses for 30min, then treated with dexamethasone or vacuole for 1h 30min, then determined using Western blot analysis. (a) NF-κB p65 protein in nuclear fraction (b) NF-κB p65 protein in cytosol fraction (c) phospho NF-κB p65 protein in whole cell fraction through Western blot. Relative ratio of NF-κB p65/p-p65 versus β-actin was measured using densitometry, and LPS was used as positive control.

**Figure S3. Effect of vacuole on the mRNA level of Anti-inflammatory cytokine such (a) IL-4 (b) IL-10 in RAW 264.7 cells stimulated by LPS.** Cells were stimulated with 1 μg/ml LPS at the indicated doses for 2 h, then treated with dexamethasone or vacuole for 22 h. The mRNA levels were measured by RT-qPCR, the gene expression levels were normalized to the expression of the housekeeping gene GAPDH

**Figure S4. Inhibitory effect of yeast vacuoles on the mRNA expression level of SASP factors (a)TNF-α, (b)IL-6, (c)iNOS in Human Lung Fibroblasts cells.** The cells were pre-treated with vacuole for 23 hours, followed by treatment with H_2_O_2_ for 1 hour. The mRNA levels were measured using RT-qPCR. The gene expression levels were normalized to the expression of the housekeeping gene β-actin

**TABLE LEGENDS**

**Table S1. Primer sequences for Real time-PCR**


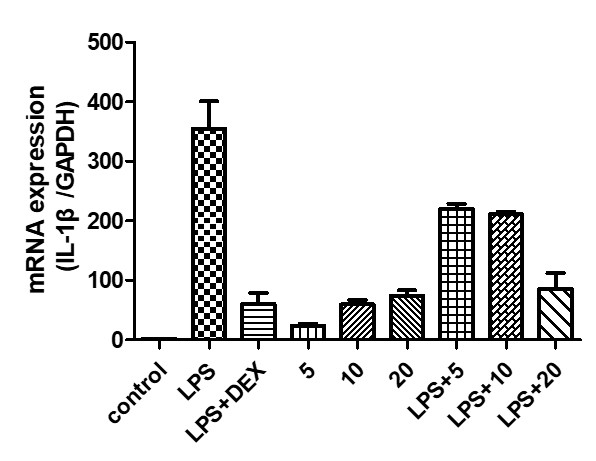


-

-

-

+

-

-

+

+

-

-

-

10

-

-

20

+

-

5

+

-

10

+

-

20

-

-

5

LPS (1 μg/mL)

Dexamethasone (1 μg/mL)

Yeast vacuole (μg/mL)

**Figure S1**


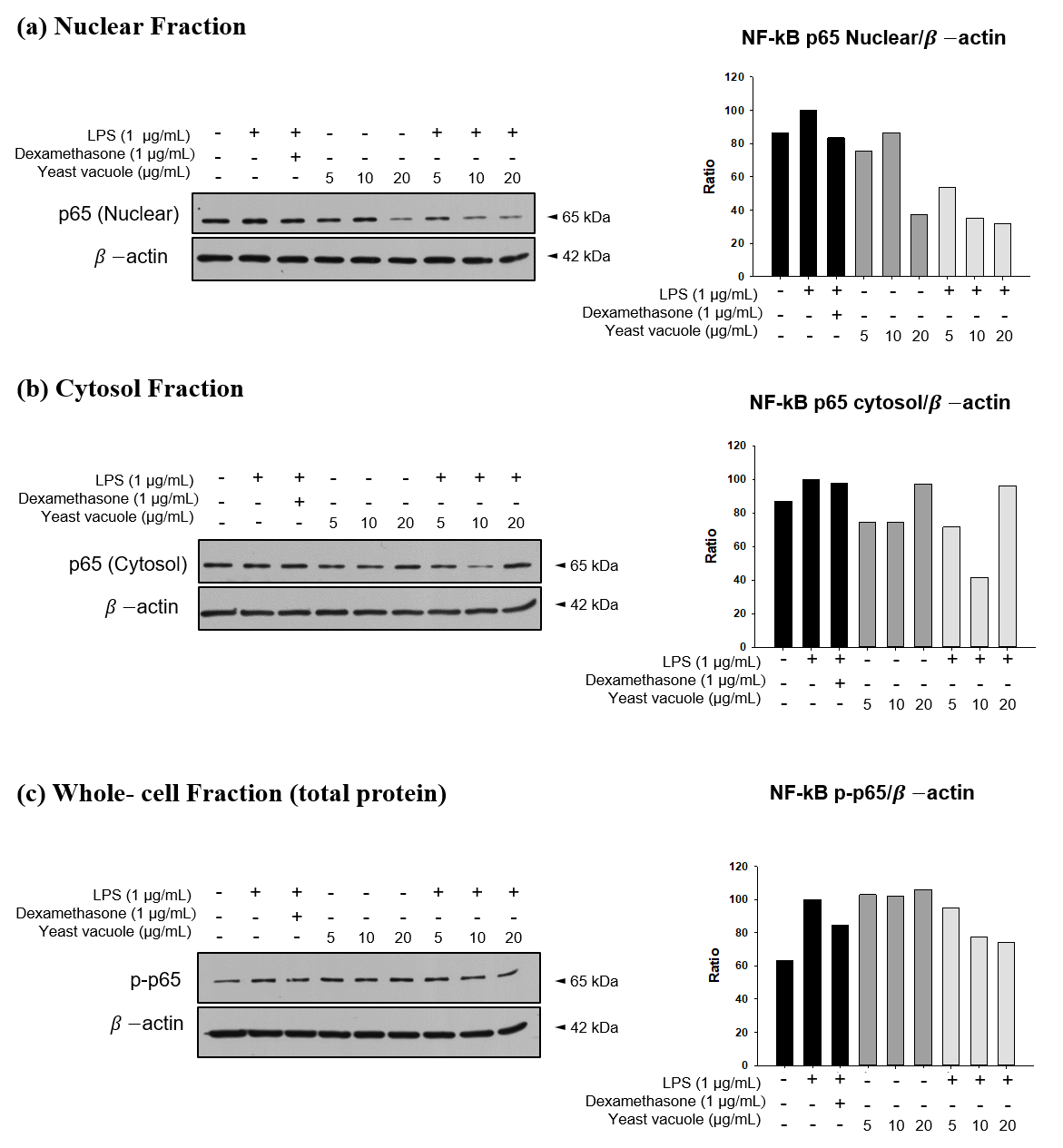


**Figure S2**

1. (b)


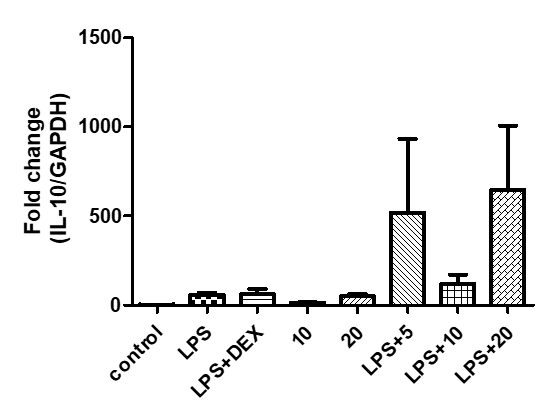


+

-

5

-

-

-

+

-

-

+

-

10

+

-

20

+

+

-

-

-

10

-

-

20

LPS (1 μg/mL)

Dexamethasone (1 μg/mL)

Yeast vacuole (μg/mL)

+

-

5

-

-

-

+

-

-

+

-

10

+

-

20

+

+

-

-

-

10

-

-

20

LPS (1 μg/mL)

Dexamethasone (1 μg/mL)

Yeast vacuole (μg/mL)

**Figure S3**

1. (b) (c)


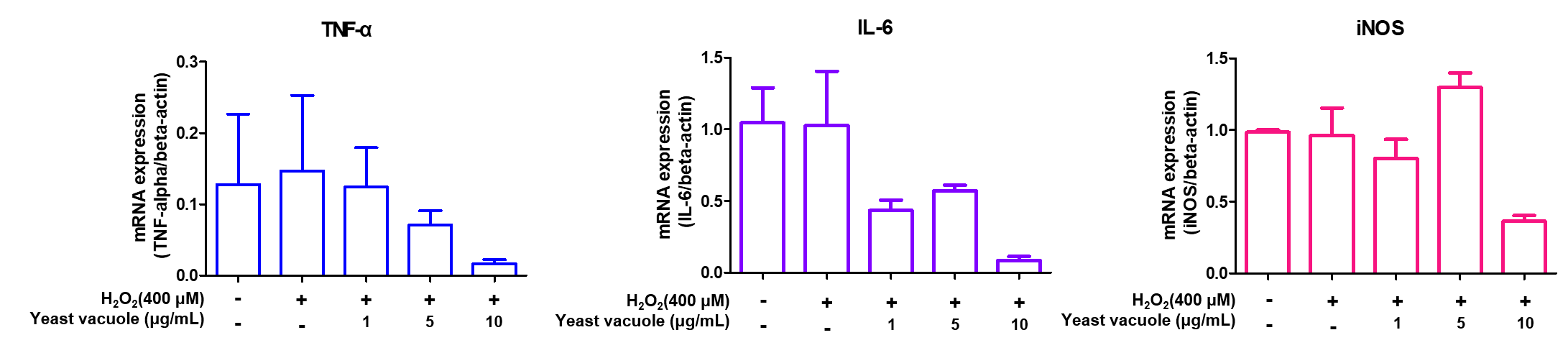


**Figure S4**

**Table S1. Primer sequences for Real time-PCR**

| **Genes** | **Forward primer sequence** | **Reverse primer sequence** |
| --- | --- | --- |
| IL-1β | 5'-AAAAGCCTCGTGCTGTCG-3' | 5'-AGGCCACAGGTATTTTGTCG-3' |
| IL-4 | 5'-TCAACCCCCAGCTAGTTGTC-3' | 5'-TGTTCTTCGTTGCTGTGAGG-3' |
| IL-10 | 5'-CCAAGCCTTATCGGAAATGA-3' | 5'-TTTTCACAGGGGAGAAATCG-3' |
| TNF-α | 5'-CAGGCGGTGCCTATGTCTC-3' | 5'-CGATCACCCCGAAGTTCAGTAG-3' |
| IL-6 | 5'-TAGTCCTTCCTACCCCAATTTCC-3' | 5'-TTGGTCCTTAGCCACTCCTTC-3' |
| iNOS | 5'-GTTCTCAGCCCAACAATACAA-3' | 5'-GTGGACGGGTCGATGTCAC-3' |
| β-actin | 5'-GGCTGTATTCCCCTCCATCG-3' | 5'-CCAGTTGGTAACAATGCCATGT-3' |
| GAPDH | 5'-CTTTGTCAAGCTCATTTCCTGG-3' | 5'-TCTTGCTCAGTGTCCTTGC-3' |
